# Targeting Lysophosphatidic Acid Signaling Retards Culture-Associated Senescence of Human Marrow Stromal Cells

**DOI:** 10.1371/journal.pone.0032185

**Published:** 2012-02-16

**Authors:** Masahiko Kanehira, Toshiaki Kikuchi, Shinya Ohkouchi, Taizou Shibahara, Naoki Tode, Arif Santoso, Hisayoshi Daito, Hiromitsu Ohta, Tsutomu Tamada, Toshihiro Nukiwa

**Affiliations:** 1 Department of Respiratory Medicine, Tohoku University Graduate School of Medicine, Sendai, Japan; 2 Department of Respiratory Medicine, Tohoku University Hospital, Sendai, Japan; Pennington Biomedical Research Center, United States of America

## Abstract

Marrow stromal cells (MSCs) isolated from mesenchymal tissues can propagate *in vitro* to some extent and differentiate into various tissue lineages to be used for cell-based therapies. Cellular senescence, which occurs readily in continual MSC culture, leads to loss of these characteristic properties, representing one of the major limitations to achieving the potential of MSCs. In this study, we investigated the effect of lysophosphatidic acid (LPA), a ubiquitous metabolite in membrane phospholipid synthesis, on the senescence program of human MSCs. We show that MSCs preferentially express the LPA receptor subtype 1, and an abrogation of the receptor engagement with the antagonistic compound Ki16425 attenuates senescence induction in continually propagated human MSCs. This anti-aging effect of Ki16425 results in extended rounds of cellular proliferation, increased clonogenic potential, and retained plasticity for osteogenic and adipogenic differentiation. Expressions of p16^Ink4a^, Rb, p53, and p21^Cip1^, which have been associated with cellular senescence, were all reduced in human MSCs by the pharmacological inhibition of LPA signaling. Disruption of this signaling pathway was accompanied by morphological changes such as cell thinning and elongation as well as actin filament deformation through decreased phosphorylation of focal adhesion kinase. Prevention of LPA receptor engagement also promoted ubiquitination-mediated c-Myc elimination in MSCs, and consequently the entry into a quiescent state, G_0_ phase, of the cell cycle. Collectively, these results highlight the potential of pharmacological intervention against LPA signaling for blunting senescence-associated loss of function characteristic of human MSCs.

## Introduction

Stem/progenitor cells are the subject of intense investigation for cell-based therapies [1]. In particular, marrow stromal cells (MSCs, also referred to as mesenchymal stem cells), which can be isolated from most mesenchymal tissues (e.g., bone marrow, fat, and blood vessels), not only represent multilineage potential for differentiating into several skeletal cell types such as osteoblasts and adipocytes but also have the capacity to secrete soluble factors that can improve repair of multiple organs such as bone, brain, heart, lung, and pancreas and modulate the immune system [2], [3], [4], [5], [6], [7], [8]. MSCs divide rapidly in culture and thus are potentially attractive for use in developing new therapeutic approaches [2], [6], [7]. However, MSCs in culture are readily observed to senesce after ≥25 population doublings, a process in which they propagate slowly, decrease their clonogenicity, and lose their potential to differentiate [7], [9], [10], [11], [12]. The propensity for a decrease in the MSC's potential prevents extensive rounds of *in vitro* expansion for obtaining clinically significant cell numbers and demands modification of culture conditions to alleviate their senescence [5], [7].

Lysophosphatidic acid (LPA) is an extracellular signaling molecule that is ubiquitously produced from membrane phospholipids through phospholipase A_2_ (PLA_2_)-mediated pathways [13], [14]. To date, five subtypes of rhodopsin-like receptors with seven-transmembrane alpha helices, LPA_1_-LPA_5_, have been reported to bind LPA and activate G proteins, thereby inducing various biological effects on diverse cellular and organ systems [13], [14]. In MSCs, some evidence has demonstrated the expression of LPA_1_-LPA_4_ receptors that are likely implicated in protecting against stress-induced apoptosis and regulating migration and differentiation [15], [16], [17], [18], [19], [20].

Here, we set out to determine whether the biological activity of LPA toward human MSCs was also associated with the phenotypic changes that MSCs entering into a state of senescence undergo during continuous *in vitro* propagation. Based on our finding that human MSCs preferentially express the LPA_1_ receptor subtype, we used a synthesized isoxazole derivative named Ki16425, 3-(4-[4-([1-(2-chlorophenyl)ethoxy]carbonyl amino)-3-methyl-5-isoxazolyl] benzylsulfanyl) propanoic acid, that antagonizes LPA binding, particularly to LPA_1_ and LPA_3_ (rank order of antagonizing affinity of Ki16425, LPA_1_≥LPA_3_≫LPA_2_) [21]. Treatment of human MSCs with Ki16425 promoted quiescence in the G_0_-phase of the cell cycle and thus enabled cells to avoid the entrance into senescence that occurs following an extended period of proliferation during continuous *in vitro* culture.

## Methods

### In vitro culture of human MSCs

Primary human MSCs were obtained at passage 1 from the Texas A&M Health Science Center for the Preparation and Distribution of Adult Stem Cells (Temple, TX). Human MSCs were obtained from three donors, 21-years-old female donor 1, 22-year-old male donor 2, and 24-year-old male donor 3; MSCs from donor 1 were used in this study unless otherwise noted. Cells were maintained at 37°C in 5% CO_2_ using complete culture medium consisting of minimum essential medium alpha (Invitrogen, Carlsbad, CA) supplemented with 17% fetal bovine serum (Nichirei, Tokyo, Japan), 100 units/ml penicillin (Invitrogen), 100 µg/ml streptomycin (Invitrogen), and 2 mM L-glutamine (Invitrogen), unless noted otherwise. To expand human MSCs, a frozen vial (passage 1, 10^6^ cells) was quickly thawed and plated in a 150-mm dish (Corning Inc., Corning, NY) and then incubated to exclude non-adherent (i.e., nonviable) cells. After 24 h, viable cells were recovered with trypsin/EDTA, re-plated at a density of 60 cells/cm^2^, and then cultured, with media replaced every 3 days. After 9 days in culture (i.e., prior to their reaching confluence), cells were harvested for passage 2 and reseeded at a density of 60 cells/cm^2^. Subsequent passages were repeated under the same conditions every 9 days for the duration of the study. Where indicated, human MSCs at passage 2 and thereafter were cultured in the presence of 5 µM Ki16425/0.1% DMSO or control vehicle alone (0.1% DMSO). The number of population doublings during a period of growth was calculated by using the formula log_10_(*Ne*/*Ns*)/log_10_2, where *Ns* is the number of cells seeded at the start, and *Ne* is the number of cells at the end of the period.

### Colony-forming unit-fibroblast (CFU-F) assay

Human MSCs were seeded into 100-mm culture dishes at 100 cells/dish. After 15 days of culture with medium replaced every 3 days, the cultures were fixed and stained with crystal violet staining solution in methanol (Kanto Chemical, Tokyo, Japan) for 20 min at RT. The dishes were washed with water and allowed to dry. Colonies were counted macroscopically, and data were reported as colony numbers per dish.

### Senescence-associated β-galactosidase (SA-β-Gal) assay

Human MSC monolayers were fixed with 0.2% glutaraldehyde for 20 min at RT, washed twice with PBS, and then stained for 24 h at 37°C with SA-β-Gal staining solution: 4 mM K_3_[Fe(CN)_6_], 4 mM K_4_[Fe(CN)_6_], 2 mM MgCl_2_, and 1 mg/ml X-gal in PBS (pH 6). Stained cells were viewed macroscopically and microscopically under bright field at 100× magnification. The total SA-β-Gal activities in the wells were also quantified with the Beta-Glo Assay System (Promega, Madison, WI) according to the manufacturer's instructions. Briefly, lysates of human MSCs were prepared from monolayers with passive lysis buffer and were then mixed with the Beta-Glo Assay Reagent. After 30 min, a luminescent signal proportional to the SA-β-Gal activity was measured for 2 s using a Luminescencer PSN luminometer (ATTO, Tokyo, Japan).

### Evaluation of telomere length

Mean telomere lengths of human MSCs were assessed in genomic DNA by real-time PCR, as described elsewhere [22]. Real-time PCR was performed in a DNA Engine Opticon 2 System (Bio-Rad Laboratories, Hercules, CA) using SYBR GreenER qPCR SuperMix Universal (Invitrogen) and two primer pairs for telomere and 36B4 (single-copy gene): telomere, 5′-GGTTTTTGAGGGTGAGGGTGAGGGTGAGGGTGAGGGT-3′ and 5′-TCCCGACTATCCCTATCCCTATCCCTATCCCTATCCCTA-3′; 36B4, 5′-CAGCAAGTGGGAAGGTGTAATCC-3′ and 5′-CCCATTCTATCATCAACGGGTACAA-3′. The telomere/36B4 ratio for the amount of the PCR product was measured as proportional to the mean telomere length, and the factor by which the telomere/36B4 ratio of the sample differed from the mean of control samples was determined as the relative telomere length.

### Western blotting

Human MSCs were lysed in RIPA buffer containing protease inhibitor cocktail and phosphatase inhibitor cocktail (all from Sigma-Aldrich, St. Louis, MO). Proteins were separated by SDS-PAGE (Novex 10% Tris-glycine) and transferred onto PVDF using iBlot (all from Invitrogen). Membranes were then blocked with 2.5% milk in PBS containing 0.05% Tween-20, immunoblotted with specific primary antibody and the relevant horseradish peroxidase (HRP)-conjugated secondary antibodies (Santa Cruz Biotechnology, Santa Cruz, CA). The signals were visualized using the ECL detection system (GE Healthcare, Piscataway, NJ). The primary antibodies used in this study were anti-phosphorylated cPLA_2_ (Ser505), anti-total cPLA_2_, anti-phosphorylated Akt (Ser473), and anti-total Akt (all from Cell Signaling Technology, Danvers, MA); anti-c-Myc (Santa Cruz Biotechnology); anti-Fbw7 and anti-p16^INK4a^ (both from Abnova, Walnut, CA); anti-Rb (retinoblastoma-associated protein/RB1, Acris Antibodies, San Diego, CA); anti-p53 (Thermo Fisher Scientific, Waltham, MA); anti-phosphorylated FAK (Tyr861) and anti-p21^Cip1^ (both from Signalway Antibody, Pearland, TX); anti-total FAK (BioLegend, San Diego, CA); and anti-β-actin (clone AC-15, Sigma-Aldrich). Quantification of band intensity was performed using Quantity One software (Bio-Rad Laboratories, Hercules, CA).

### Reverse transcriptase–polymerase chain reaction (RT-PCR)

Total RNA was extracted using an RNeasy Plus kit (QIAGEN, Valencia, CA). RNA was reverse-transcribed using the SuperScript III first-strand synthesis system (Invitrogen) to generate cDNA. The resulting cDNA was amplified by semiquantitative PCR using Platinum *Taq* DNA polymerase (Invitrogen) or by real-time PCR as described above. All real-time PCR mRNA expression data were normalized to glyceraldehyde-3-phosphate dehydrogenase (GAPDH) expression, and the factor by which the normalized expression of the sample differed from the mean of control or LPA_1_ samples was determined as the relative gene expression. The primer pairs used in this study were as follows; LPA_1_, 5′-AATCGGGGATACCATGATGAGTCTT-3′ and 5′-CCAGGAGTCCAGCAGATGATAAA-3′; LPA_2_, 5′-CGCTCAGCCTGGTCAAGACT-3′ and 5′-TTGCAGGACTCACAGCCTAAAC-3′; LPA_3_, 5′-AGGACACCCATGAAGCTAATGAA-3′ and 5′-GCCGTCGAGGAGCAGAAC-3′; LPA_4_, 5′-AAAGATCATGTACCCAATCACCTT-3′ and 5′-CTTAAACAGGGACTCCATTCTGAT-3′; LPA_5_, 5′-CTCTCCTACTACGCACTGCACCACT-3′ and 5′-GAAGCTCTCGAAGCATAGGCGCA-3′; Osteopontin, 5′-CTAGGCATCACCTGTGCCATACC-3′ and 5′-CAGTGACCAGTTCATCAGATTCATC-3′; FABP4, 5′-ATGGGATGGAAAATCAACCA-3′ and 5′-GTGGAAGTGACGCCTTTCAT-3′; GAPDH, 5′-GAGTCAACGGATTTGGTCGT-3′ and 5′-TTGATTTTGGAGGGATCTCG-3′.

### Fluorescent staining and imaging

Human MSCs treated with or without Ki16425 were fixed with 4% paraformaldehyde for 30 min, washed with PBS, and incubated for 30 min with 5 U/ml fluorescein isothiocyanate (FITC)-conjugated phalloidin (a high-affinity filamentous actin probe; Enzo Life Sciences, Farmingdale, NY), 5 µg/ml propidium iodide (nuclear co-staining), and 0.1% Tween-20 in PBS. Stained cells were viewed at 100× magnification using a BX51 fluorescent microscopy (Olympus, Tokyo, Japan), and the cell morphology was monitored.

### Cell cycle assay

Human MSCs were collected and fixed with 80% ethanol overnight at 4°C. Fixed cells were then resuspended in PBS containing 0.1% bovine serum albumin and 0.25 µg/ml 7-AAD (DNA dye; Imgenex, San Diego, CA). After incubation at 4°C for 1 h, pyronin Y (RNA dye; Sigma-Aldrich) was added to a final concentration of 2 µg/ml, and cells were incubated for a further 1 h and then maintained at 4°C. G_0_ and G_1_ cell populations were identified as displaying low and high RNA staining, respectively, within the G_0_/G_1_ DNA phase by an EPICS XL cytometer with EXPO32 ADC software (Beckman Coulter, Miami, FL).

### Osteogenic and adipogenic differentiation assays

Human MSCs were seeded in six-well Collagen I Cellware plates (BD Biosciences) with osteogenic medium (complete culture medium, 1 nM dexamethasone, 20 mM β-glycerophosphate, and 50 µg/ml ascorbate 2-phosphate) or adipogenic medium (complete culture medium, 0.5 µM dexamethasone, 0.5 µM isobutylmethylxanthine, and 50 µM indomethacin). Plates were incubated for 2 (osteogenic) or 3 (adipogenic) weeks with medium replenishment every 3 days. After the differentiation process, cell monolayers were fixed with 10% formaldehyde for 15 min and stained with 0.1% alizarin red S solution (osteogenesis, Wako Chemicals, Richmond, VA) for 20 min or with 0.18% oil red O (adipogenesis, Sigma-Aldrich) in 60% isopropanol for 20 min. Stained cells were viewed macroscopically and microscopically under bright field at 100× magnification. To quantify staining intensity, the dye remaining in each well was extracted by lysing the cells in 0.5 ml of passive lysis buffer (Promega). The absorbance of the solution was then measured at 570 nm using an EMax microplate reader (Molecular Devices, Sunnyvale, CA).

### Statistical analysis

All data are reported as means ± standard error unless otherwise stated. Statistical comparisons were performed by two-tailed Student's *t*-test. In all analyses, *P*<0.05 was taken to indicate statistical significance.

## Results

### Senescence-associated impairment of MSC clonogenic potential in continual culture

**Figure 1:**
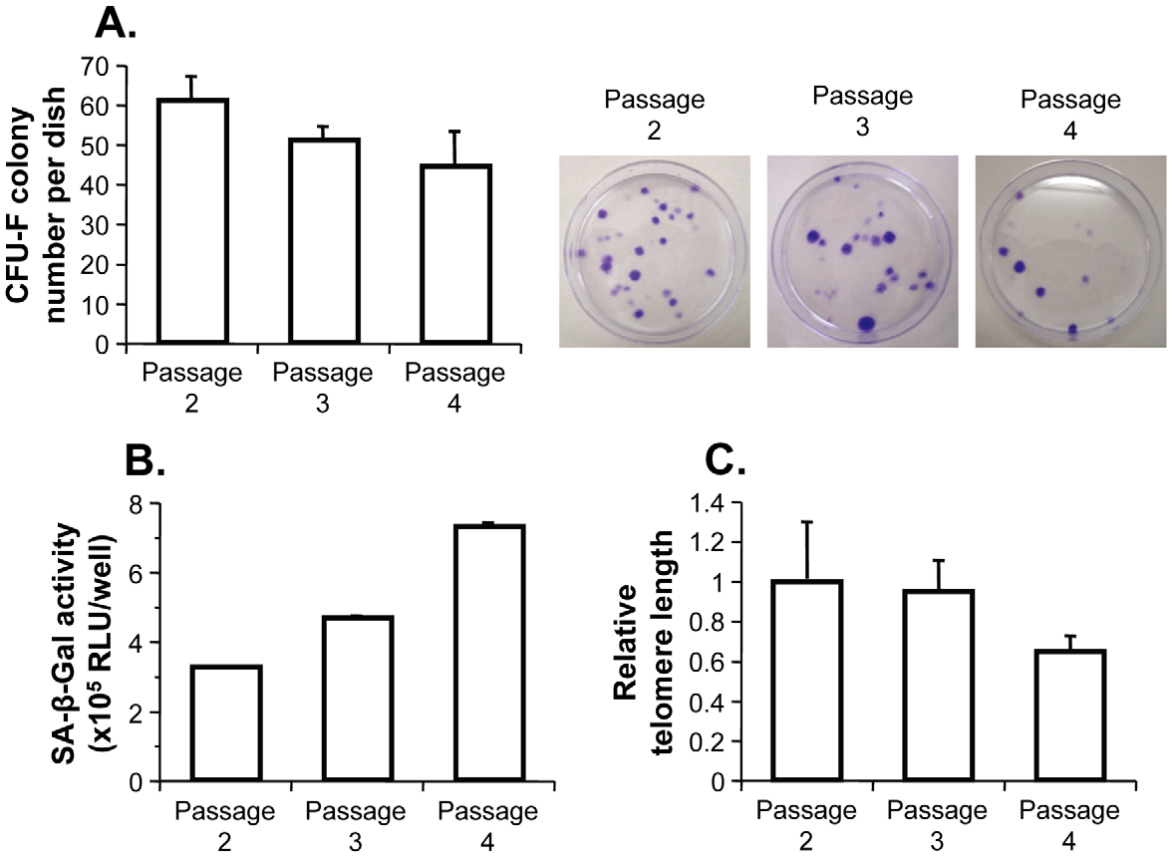
In vitro continuous culture of human MSCs reduced their clonogenic capacity with cellular senescence over three consecutive passages at passages 2, 3, and 4. **A.** CFU-F assay. CFU-F colonies from human MSCs (passages 2, 3, and 4; 100 cells) were counted after culture for 15 days. On the right side are shown representative CFU-F colonies stained with crystal violet. **B.** SA-β-Gal assay. The total SA-β-Gal activities of human MSCs (passages 2, 3, and 4) were quantified in the wells of six-well plates as the luminescent intensity (relative luminescence units, RLU) that was generated due to the cleavage of a luciferin-galactoside substrate. **C.** Telomere measurement. Telomere lengths were determined in human MSCs (passages 2, 3, and 4) by real-time PCR and quantified relative to the mean of passage 2. For all panels, the data are presented as the means ± standard error (*n* = 3).

To ensure that the functional capacity of MSCs was reduced during cultivation *in vitro*, we examined the colony-forming efficiency, cellular senescence, and telomere length of human MSCs with increasing cell passages (**Fig. 1**). The number of MSCs capable of initiating colonies (the colony-forming unit fibroblasts, CFU-Fs) is commonly used as a measure of differential potential and self-renewal capacity [9]. The frequency of CFU-Fs was markedly decreased during cell passages 2–4, although the CFU-F numbers of passages 3 and 4 were not significantly different from that of passage 2 (passage 3, *P* = 0.06; passage 4, *P* = 0.05; **Fig. 1A**). The senescent state in continuous MSC cultures was assessed using senescence-associated β-galactosidase (SA-β-Gal), an established biomarker associated with cellular aging [23], [24], [25]. Human MSCs at passages 2–4 showed a gradual increase in SA-β-Gal activity; these activities were significantly different (passages 2–3, *P*<0.01; passages 3–4, *P*<0.01; passages 2–4, *P*<0.01; **Fig. 1B**). The gradual induction of cellular senescence in continuous MSC culture was inversely correlated with telomere length; this decreased significantly between passages 3 and 4 (*P*<0.05; **Fig. 1C**). These data show that human MSCs during culture under standard conditions undergo a senescent change as a consequence of telomere shortening, accompanied by a reduction in colony-forming efficacy.

### An LPA receptor antagonist reduces MSC senescent changes

**Figure 2:**
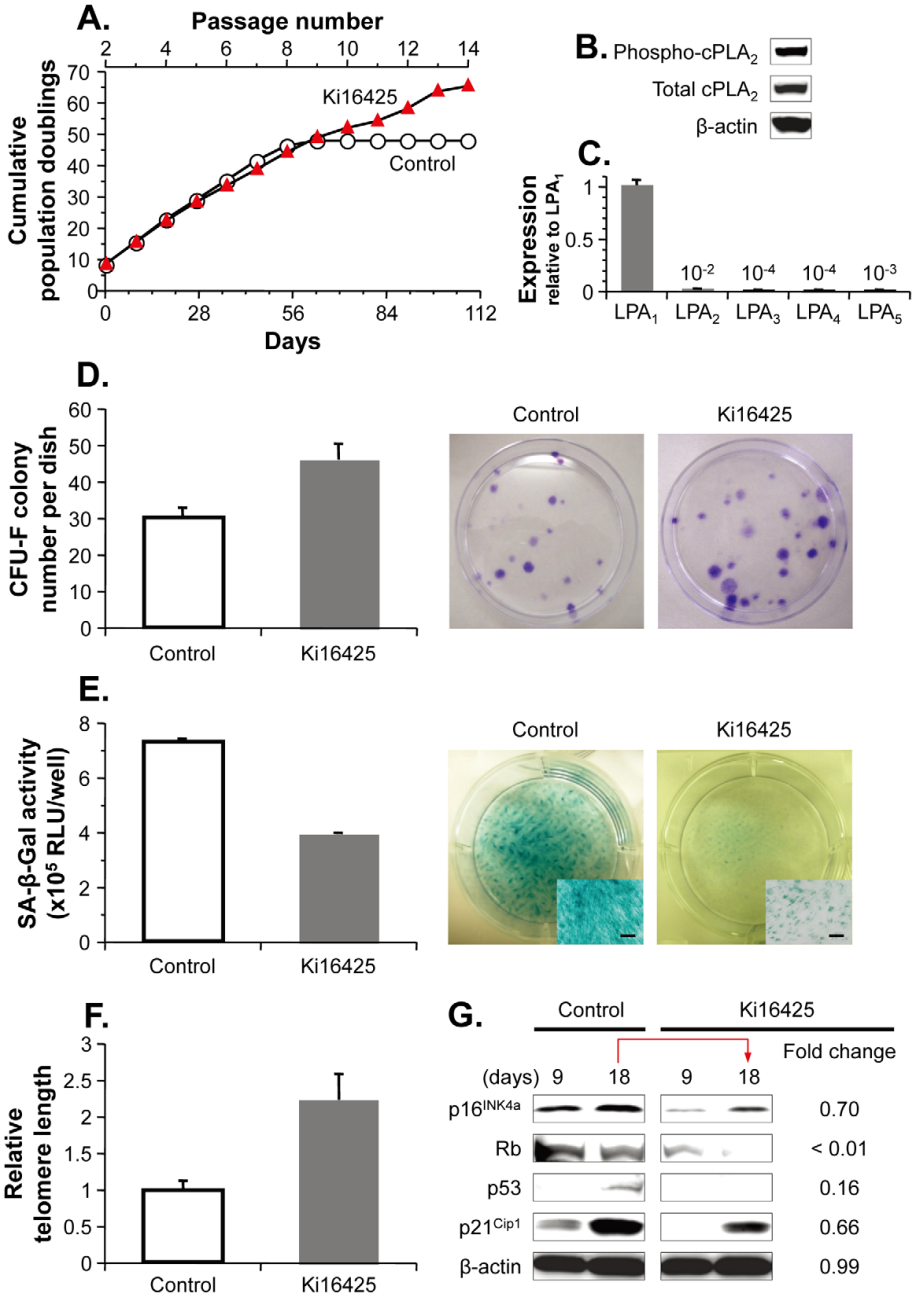
Decreased self-renewal capacity associated with senescence was prevented in human MSCs following treatment with Ki16425, an LPA_1_/LPA_3_ antagonist. **A.** Growth kinetics during serial passage. Human MSCs at passage 2 (8.1 population doublings) were serially passaged every 9 days in the presence or absence of Ki16425. Cumulative population doublings are presented as the means of duplicates. **B.** Western blotting analysis of total and phosphorylated cPLA2 in human MSCs at passage 2. **C.** Real-time PCR analysis of LPA receptor gene expression in human MSCs at passage 2. Levels of mRNA were quantified relative to the mean of LPA_1_ samples. **D.** CFU-F assay. Human MSCs at passage 2 were cultured in the presence or absence of Ki16425 for two additional passages (27 days). CFU-F colonies initiated from the treated cells (passage 5, 100 cells) were counted after 15 days of normal culture. On the right side are shown representative CFU-F colonies stained with crystal violet. **E.** SA-β-Gal assay. The total SA-β-Gal activities of Ki16425- and vehicle-treated human MSCs were quantified in the wells of six-well plates as the luminescent intensity (relative luminescence units, RLU). On the right side are shown representative human MSCs stained for SA-β-Gal. Scale bars in inset boxes, 200 µm. **F.** Telomere measurement. Telomere lengths were determined in Ki16425- and vehicle-treated human MSCs by real-time PCR and quantified relative to the mean of vehicle controls. **G.** Western blotting analysis of cell-cycle components. Human MSCs at passage 2 were cultured in the presence or absence of Ki16425 for the indicated days prior to cell lysis. Fold-change represents decrease in band intensity of Ki16425 treatment for 18 days compared with a control treatment for the same period of time. For panels **C**, **D**, **E**, and **F**, the data are presented as the means ± standard error (*n* = 3).

We next investigated the role of lysophosphatidic acid signaling in shaping the cell growth kinetics of continuously-cultured human MSCs. Under standard culture conditions, human MSCs exhibited a relatively constant population doubling rate for 54 days, i.e., six passages, after which they entered growth arrest, with no appreciable increase in cumulative population doublings (**Fig. 2A**). In contrast, upon addition of Ki16425, a selective antagonist for lysophosphatidic acid receptor subtypes 1 and 3 (LPA_1_ and LPA_3_), human MSCs maintained their proliferative potential over the period studied, and cumulative population doublings increased at a constant rate for 108 days, i.e., 12 passages (**Fig. 2A**). Immunoblotting of human MSC lysates revealed Ser505 phosphorylation and activation of cytosolic phospholipase A_2_ (cPLA_2_), a major enzyme producing lysophosphatidic acid from membrane phospholipids (**Fig. 2B**) [26]. Human MSCs also expressed the lysophosphatidic acid LPA_1_ receptor gene at much higher levels relative to LPA_2_, LPA_3_, LPA_4_, and LPA_5_ receptor genes, as determined by real-time PCR analysis (*P*<0.001 compared with all other LPA receptors, **Fig. 2C**). The expression level of the LPA_1_ receptor gene was stable in human MSCs cultured for up to 54 days (not shown). These data suggest that Ki16425 facilitates extended MSC expansion by hindering autocrine and paracrine lysophosphatidic acid signaling mainly through the LPA_1_ receptor.

Expansion of somatic diploid cells is generally regulated by replicative senescence due to telomere shortening during cell division [23], [25]. We therefore examined the impact of Ki16425 on cellular functions associated with the state of senescence. Antagonism of lysophosphatidic acid LPA_1_/LPA_3_ receptors in human MSC cultures resulted in a striking cellular phenotype, with induction of clonogenic capacity with elevated CFU-F numbers (*P*<0.01; **Fig. 2D**). Furthermore, whereas a majority of MSCs in control cultures showed strong SA-β-Gal staining, few of the cells treated with Ki16425 showed staining coincident with the significantly reduced activity of SA-β-Gal (*P*<0.001; **Fig. 2E**). The telomere length of human MSCs cultured with Ki16425 was 2.2-fold greater than that of control MSCs (*P*<0.005; **Fig. 2F**). Similar results were achieved in human MSCs from donors of different age (**Fig. S1**).

Activation of the p16^Ink4a^-Rb and p53-p21^Cip1^ signaling pathways has been shown to reduce stem/progenitor-cell frequency and function in a variety of aging tissues [27], [28], [29]. We therefore investigated the potential of Ki16425 to decrease protein levels of components in these signaling cascades. Immunoblotting of total cell lysates revealed reduced expression of p16^Ink4a^, Rb, p53, and p21^Cip1^ in Ki16425-treated human MSCs compared with that in time-point-matched control MSCs; no apparent differences in confluency of the MSC culture were observed between Ki16425 and control treatments (Fig. 2G). The upward trend evident at 9–18 days was reduced by Ki16425 treatment, with the exception of Rb, in which case a downward trend was accelerated (**Fig. 2G**). Taken together, these data suggest that lysophosphatidic acid signaling through the LPA_1_/LPA_3_ receptors is essential for regulation of the functional properties of human MSCs. Furthermore, during continuous propagation, LPA receptor self-activation leads to induction of cellular senescence.

### Inhibition of LPA signaling results in alterations in cell shape and transition to a quiescent state

**Figure 3:**
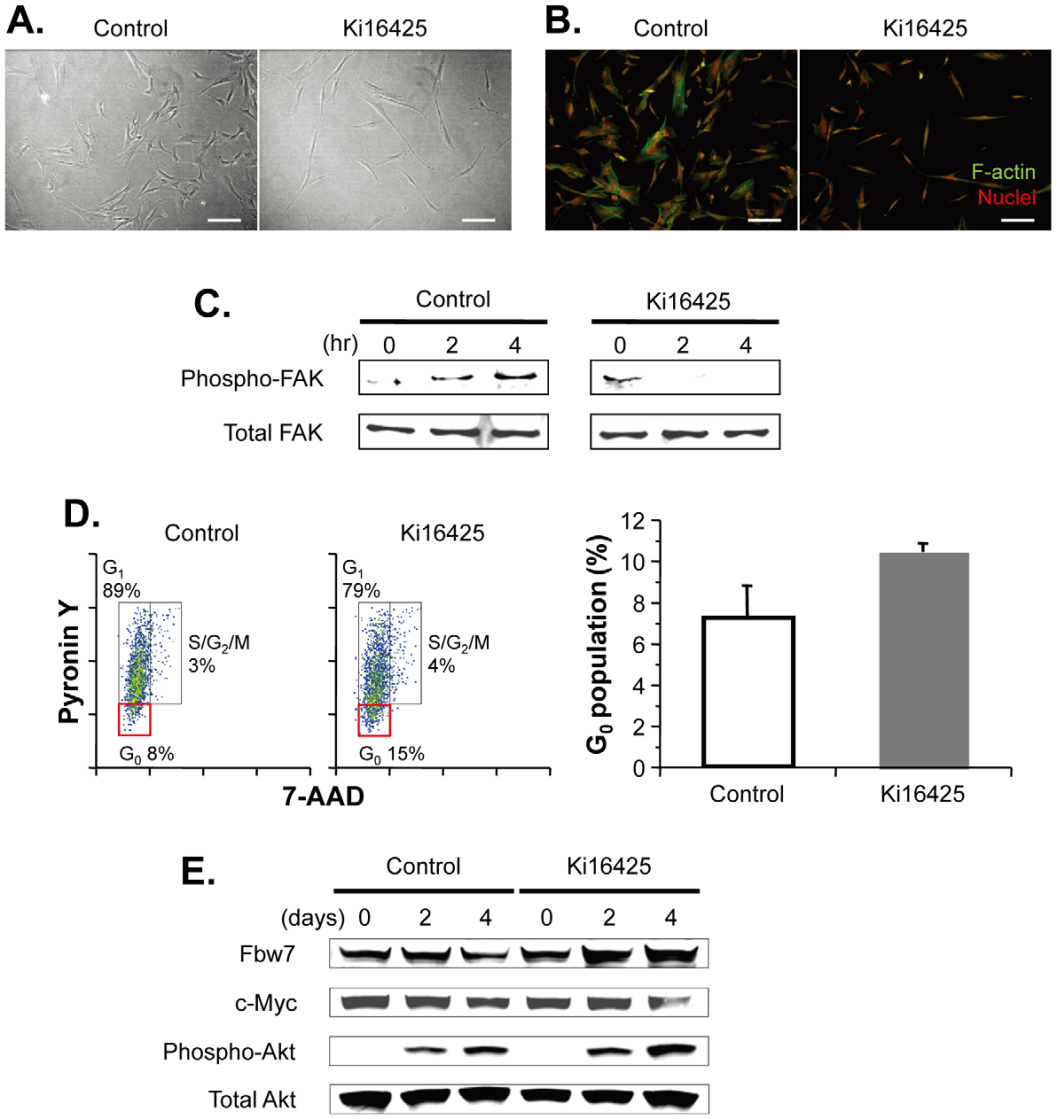
LPA_1/3_ inhibition of human MSCs reduced actin polymerization and increased cell-cycle quiescence. **A, B.** Phenotypic characteristics of human MSCs treated with Ki16425 or vehicle alone for 48 h after plating. Shown are phase-contrast images (panel **A**) and fluorescent images in which filamentous actin (F-actin) was visualized with green phalloidin-FITC staining and nuclei were stained with red propidium iodide (panel **B**). Scale bars, 200 µm. **C.** Western blotting analysis to evaluate the phosphorylation and activation status of focal adhesion kinase (FAK). **D.** Cell-cycle analysis. Human MSCs treated with Ki16425 or vehicle alone for 72 h were fixed and then stained for DNA and RNA with 7-AAD and pyronin Y, respectively. Their cell-cycle status was assessed based on their DNA and RNA content by flow cytometry. A representative of three experiments is shown on the left side, and the bar graph summarizes the results of the G_0_ proportion on the right side. The data are presented as the means ± standard error (*n* = 3). **E.** Western blotting analysis of signaling molecules associated with the Akt pathway. For panels **C** and **E**, human MSCs were cultured in the presence or absence of Ki16425 for the indicated times prior to cell lysis.

Morphological changes in human MSCs likely reflect cellular function, as has been reported [30], [31], [32], [33]. We then assessed cell morphology by phase-contrast microscopy and found that human MSCs adopted a thin and elongated phenotype upon treatment with Ki16425, an LPA_1_/LPA_3_ receptor antagonist, compared with controls (**Fig. 3A**). This phenotypic change correlates with alteration of cytoskeleton content; immunostaining for filamentous actin (F-actin) after disruption of LPA_1_/LPA_3_ receptors with Ki16425 revealed deformation of actin filaments running through the cell body of human MSCs when compared to the control (**Fig. 3B**). As analyzed by Western blotting of cell extracts from human MSCs, phosphorylation of focal adhesion kinase (FAK), a key signaling event implicated in actin organization through Rho activation, was attenuated in the hours immediately following Ki16425 treatment (**Fig. 3C**) [34], [35].

We next asked whether LPA receptor-selective inhibition of human MSCs may also affect the cell-cycle state. Previous studies have shown a link between cell shape and proliferation kinetics, indicating the limited proliferative capacity of thin spindle-shaped cells such as Ki16425-treated MSCs [30], [32]. As anticipated, cell-cycle analysis of human MSCs showed that Ki16425-treated cells were significantly enriched for pyronin Y^low^-staining cells in the G_0_ phase compared with control cells (*P*<0.05; **Fig. 3D**). Enrichment of the G_0_ phase MSC population by Ki16425 suggests that in the absence of LPA_1/3_ receptor-mediated signaling, human MSCs are predisposed to exit from the cell cycle and enter a quiescent state. To further characterize the nature of cell-cycle regulation, we analyzed expression of the transcription factor c-Myc, a key component of the cell-cycle transition, in human MSCs that had been treated with Ki16425 for either 2 or 4 days and found that it was lower at the later time point (i.e., 4 days) compared with control cells (**Fig. 3E**). This reduced c-Myc level in Ki16425-treated MSCs was accompanied by an increase in expression of FBW7 (F-box and WD repeat domain-containing 7), a substrate recognition component of SCF-type ubiquitin ligase that targets c-Myc and mediates polyubiquitination for proteasomal degradation (**Fig. 3E**) [36], [37]. Paradoxically, the results of immunostaining also showed that levels of phosphorylated Akt (Phospho-Akt), which may increase c-Myc protein levels by inhibiting GSK3 (glycogen synthase kinase 3) and/or activating eIF4E (eukaryotic initiation factor 4E), became gradually elevated in human MSCs upon Ki16425 treatment compared with time-point-matched controls (**Fig. 3E**) [38], [39]. Besides c-Myc accumulation, a variety of signaling molecules downstream of Akt have been reported to promote cell survival, growth, and proliferation, suggesting that disruption of LPA_1_/LPA_3_ receptor engagement coordinates several signal pathways in MSCs and induces them to enter a cell-cycle state of quiescence without impairing cell growth kinetics.

### Absence of LPA receptor engagement potentiates MSC differentiation capacity

**Figure 4:**
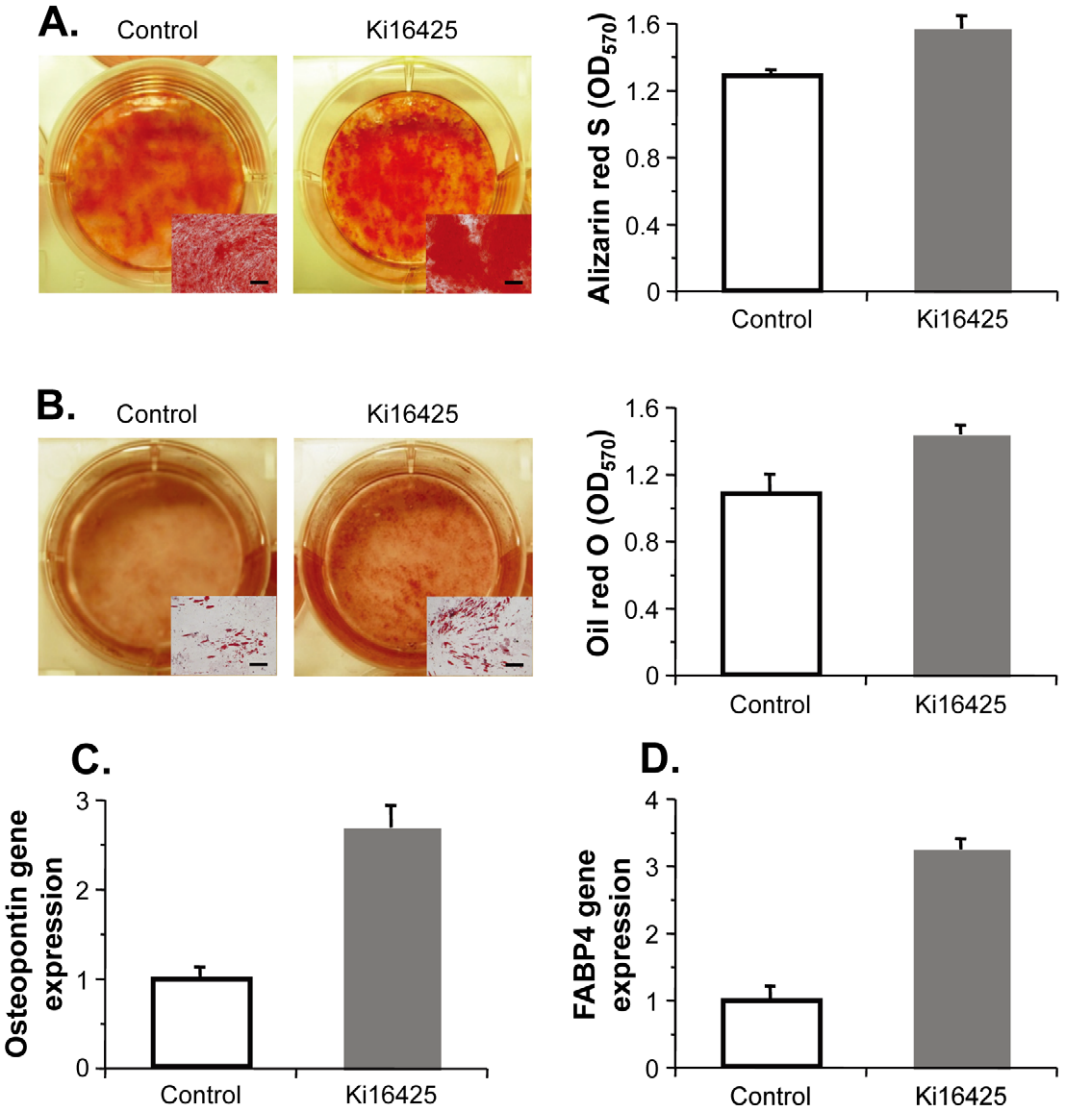
Maintenance of osteogenic and adipogenic potency in human MSCs was promoted through impaired LPA_1/3_ signaling. Human MSCs were cultured for 6 days in the presence of Ki16425 or vehicle alone and subjected to osteogenic (panels **A** and **C**) or adipogenic (panels **B** and **D**) induction for 2 or 3 weeks, respectively. **A.** Osteogenic cultures were stained with alizarin red S. **B.** Adipogenic cultures were stained with oil red O. For panels **A** and **B**, the staining was quantified as absorbance at OD_570_ per well (right side). Scale bars in inset boxes (left side), 200 µm. **C.** Osteopontin gene expression in osteogenic cultures. **D.** Fatty acid-binding protein 4 (FABP4) gene expression in adipogenic cultures. For panels **C** and **D**, mRNA levels were determined by real-time PCR and quantified relative to the mean of vehicle controls. For all panels, the data are presented as the means ± standard error (*n* = 3).

To examine whether the differentiation potential of the human MSCs was altered when LPA_1_/LPA_3_ receptor-mediated signaling was crippled, we undertook a study assaying differentiation into osteoblasts and adipocytes. After osteogenic culture, alizarin red S staining revealed that Ki16425-treated MSCs had calcium phosphate mineralization to a higher degree than did control MSCs, and there was also a significant difference in the amount of extracted dye (*P*<0.005; **Fig. 4A**). On the other hand, when subjected to adipogenic differentiation, Ki16425-treated MSCs exhibited a higher level of oil red O staining (an indicator of intracellular lipid accumulation), with a concomitant increase in the quantity of dye extracted (*P*<0.005; **Fig. 4B**). This greater potential to differentiate into osteoblasts and adipocytes is further supported by the finding that under each differentiation-inducing culture, the proper gene expression was enhanced in Ki16425-treated MSCs. As determined by real-time PCR, the expression of genes encoding osteopontin or fatty acid-binding protein 4 (FABP4) was significantly upregulated in human MSCs that had been exposed to Ki16425 before osteogenic or adipogenic differentiation, respectively (osteopontin, *P*<0.001, **Fig. 4C**; FABP4, *P*<0.001, **Fig. 4D**).

## Discussion

In this study, we demonstrated that LPA plays a prominent role in the induction of cellular senescence that human MSCs undergo through continual propagation, as evidenced by attenuation of senescence-associated changes in human MSCs treated with Ki16425, an LPA receptor engagement antagonist. The Ki16425 treatment of human MSCs reduced both SA-β-Gal accumulation and telomere shortening through the inactivation the p16^Ink4a^-Rb and p53-p21^Cip1^ signaling pathways, resulting in extensive propagation with retained clonogenic and differentiation potential. Besides the functional relevance, the anti-aging effects of the Ki16425 treatment were accompanied by morphological changes that were caused by dephosphorylation of focal adhesion kinase via preventing polymerization of actin filaments. LPA-associated processes also involve cell-cycle regulation in human MSCs, and therefore the targeting treatment increased quiescent MSCs in the G_0_ phase of the cell cycle as a consequence of promoting ubiquitin-mediated c-Myc degradation.

MSCs possess the potential not only to differentiate into a variety of mesenchymal lineages such as osteoblasts and adipocytes, but also to secrete both defined and as-yet-undefined paracrine soluble factors that may ameliorate several clinical disorders including myocardial infarction, diabetes, sepsis, hepatic failure, acute renal failure, and acute lung injury [4], [6], [40], [41], [42], [43], [44]. This property makes MSCs attractive for the cell-based therapeutic approach because they can be easily isolated from human mesenchymal tissues and subsequently expanded *in vitro* for administration [6], [40], [41], [42]. However, previous studies have shown that human MSCs tend to enter a state of senescence under standard culture conditions as early as 25 population doublings, which poses a major stumbling block to *in vitro* MSC propagation while retaining therapeutic potential [7], [9], [10], [45], [46]. In this study, MSCs cultured in the standard conditions also exhibited signs of senescence at early passage and thereafter entered a state of arrested growth, probably due to the disparity between cell senescence and the replicative capacity [47].

Cellular senescence conceptually consists of two categories, replicative and stress-induced [23], [25], [48]. Whereas replicative senescence is attributed to critical attrition of the telomere that counts the number of cell divisions by shortening its length with every division, stress-induced senescence is viewed as occurring without telomere shortening in normal cells exposed to various physical stresses, such as DNA-damaging agents, oxidative stress, and metabolic perturbations [23], [25], [49], [50]. The conditions that induce these two responses cannot always be distinguished. For example, some types of normal cells exhibit replicative senescence due to the cumulative stress of certain culture conditions that are physiologically stressful to the cells [23]. This knowledge leads us to attempt modified culture conditions in which human MSCs undergo senescence-retarding expansion. Several pieces of evidence in the present study substantiate that this effort is valid. Supplement of standard culture conditions with an LPA receptor antagonist prevented telomere shortening of human MSCs and consequently brought about extensive expansion of MSCs, with preservation of their CFU-F-forming and differentiation capacity.

Over the past few years great interest has been shown in LPA, a water-soluble phospholipid, not only because it is an inert metabolite in the biosynthesis of membrane phospholipids but also because it is an important signaling molecule [13], [14], [51]. Cellular responses altered by LPA include a diverse range of mammalian cell processes that are mediated by five G protein-coupled receptors, LPA_1–5_ [13], [14]. Human MSCs have been reported to express the LPA_1_, LPA_2_, LPA_3_, and LPA_4_ receptors, and we have shown that LPA_1_ expression is markedly higher than that of the others [16], [20]. This observation suggests that the functional role of Ki16425, a selective antagonist for both LPA_1_ and LPA_3_ receptors, is probably mediated by disturbance of LPA_1_-receptor engagement on human MSCs.

LPA receptors have broad expression patterns that allow LPA to exert biological effects on many different target tissues [13], [14]. Although some of the mechanisms regulating stem-cell functions are now beginning to be clarified, much remains unknown [14]. In this context, some reports regarding LPA-mediated MSC regulation have been published. First, Jaganathan, *et al.* and Lee, *et al.* showed that LPA treatment of human MSCs activated intracellular Rho and increased actin stress fibers, consistent with our finding that repression of LPA signaling by Ki16425 decreased actin polymerization of human MSCs probably due to Rho inactivation [16], [17]. Second, Chen, *et al.* demonstrated that LPA protected rat MSCs against apoptosis induced by cellular stresses such as hypoxia, serum deprivation, and ischemia. Contrary to their findings, the present study indicates that targeting endogenous LPA signaling through autocrine and/or paracrine mechanisms extends the lifespan of human MSCs over 112 population doublings rather than promoting apoptosis, at least under steady-state culture conditions [15], [18], [19]. Third, Liu, *et al.* pointed out that inhibition of LPA signaling with Ki16425 during osteogenic differentiation abrogated the osteogenesis of human MSCs over-expressing telomerase. This is contrary to our finding that pretreatment with Ki16425 induced osteogenesis of human MSCs [20]. The inconsistency of these findings may be explained by an over-expression of telomerase in human MSCs or LPA signaling status under osteogenic culture conditions. Finally, Mansell, *et al.* treated human MSCs *in vitro* with albumin-bound LPA and vitamin D3, finding that the treatment co-operatively promoted the osteoblastogenesis [52]. Understanding the precise role of LPA signaling in MSC osteogenic differentiation as well as the relevance of these *in vitro* observations to *in vivo* cellular function thus awaits further studies.

## Supporting Information

Figure S1 **Prevention of decreased self-renewal capacity associated with senescence was also observed in Ki16425-treated human MSCs from different donors.** **A.**, **B.** CFU-F assay. Human MSCs from donor 2 (A) or donor 3 (B) at passage 2 were cultured in the presence or absence of Ki16425 for two additional passages (27 days). CFU-F colonies initiated from the treated cells (passage 5, 100 cells) were counted after 15 days of normal culture. **C.**, **D.** SA-β-Gal assay. The total SA-β-Gal activities of Ki16425- and vehicle-treated human MSCs from donor 2 (C) or donor 3 (D) were quantified in the wells of six-well plates as the luminescent intensity (relative luminescence units, RLU). **E.**, **F.** Telomere measurement. Telomere lengths were determined in Ki16425- and vehicle-treated human MSCs from donor 2 (E) or donor 3 (F) by real-time PCR and quantified relative to the mean of vehicle controls. For all panels, the data are presented as the means ± standard error (*n* = 3). *P* values compared to controls were indicated.
(TIF)
