# Supplementary figures and images for: Targeting Lysophosphatidic Acid Signaling Retards Culture-Associated Senescence of Human Marrow Stromal Cells
Source: PLoS One. 2012 Feb 16;7(2):e32185. doi: 10.1371/journal.pone.0032185 (PMC3281120; doi:10.1371/journal.pone.0032185)

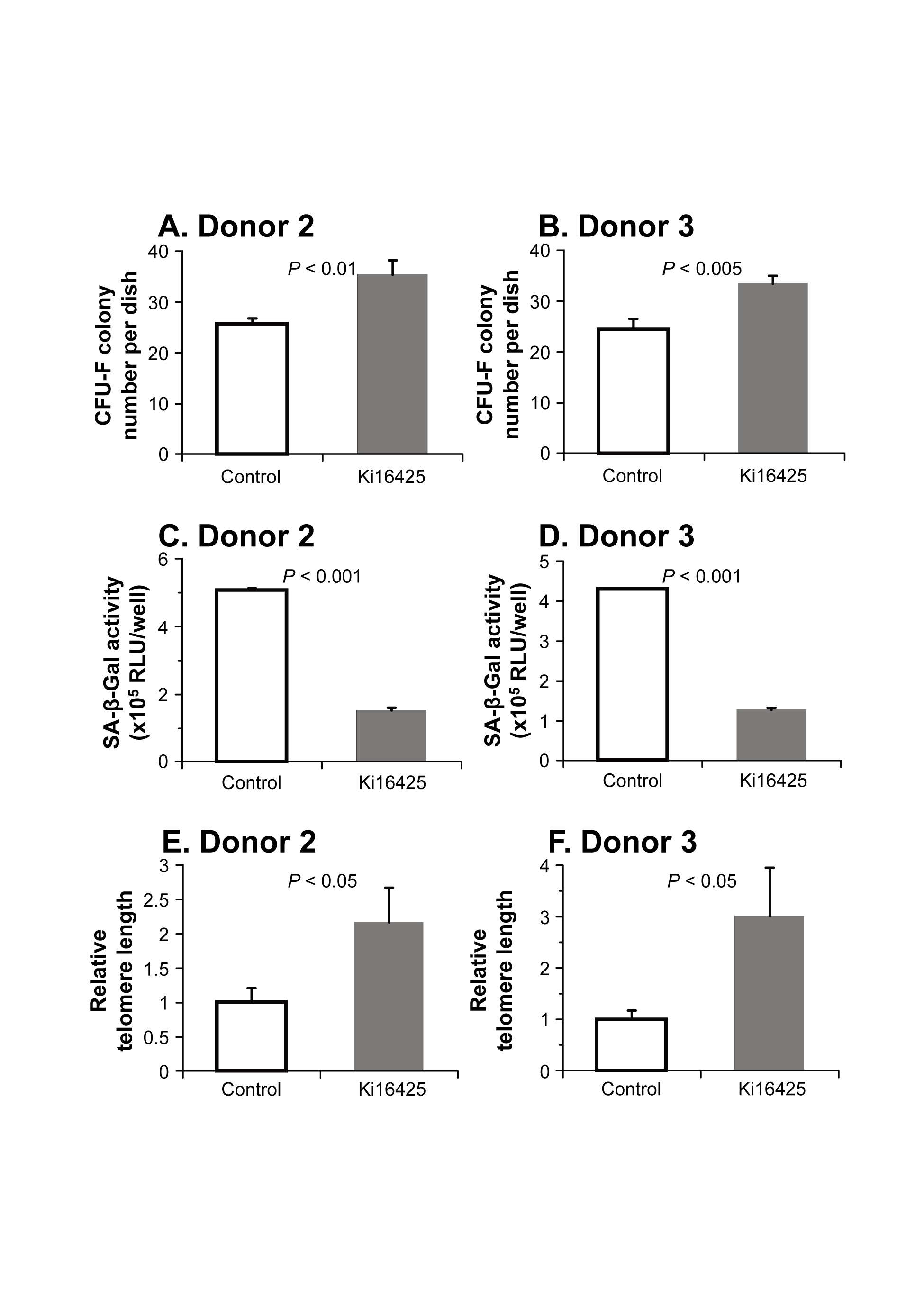

Supplement: Figure S1 — Prevention of decreased self-renewal capacity associated with senescence was also observed in Ki16425-treated human MSCs from different donors. A., B. CFU-F assay. Human MSCs from donor 2 (A) or donor 3 (B) at passage 2 were cultured in the presence or absence of Ki16425 for two additional passages (27 days). CFU-F colonies initiated from the treated cells (passage 5, 100 cells) were counted after 15 days of normal culture. C., D. SA-β-Gal assay. The total SA-β-Gal activities of Ki16425- and vehicle-treated human MSCs from donor 2 (C) or donor 3 (D) were quantified in the wells of six-well plates as the luminescent intensity (relative luminescence units, RLU). E., F. Telomere measurement. Telomere lengths were determined in Ki16425- and vehicle-treated human MSCs from donor 2 (E) or donor 3 (F) by real-time PCR and quantified relative to the mean of vehicle controls. For all panels, the data are presented as the means ± standard error (n = 3). P values compared to controls were indicated. (TIF) [file pone.0032185.s001.tif]
